# Zellkulturbasierte Influenzaimpfstoffe: eine effektive Impfstoffoption für unter 60-Jährige

**DOI:** 10.1007/s00508-024-02327-3

**Published:** 2024-02-23

**Authors:** Barbara C. Gärtner, Dietmar Beier, Gunther Gosch, Klaus Wahle, Luise Wendt, Laura-Christin Förster, Kim J. Schmidt, Tino F. Schwarz

**Affiliations:** 1 Institut für Medizinische Mikrobiologie und Hygiene, Universitätsklinikum des Saarlandes, Homburg/Saar, Deutschland; 2 Sächsische Impfkommission, Chemnitz, Deutschland; 3 Kinderarztpraxis am Domplatz, Magdeburg, Deutschland; 4 Medizinische Fakultät, Universität Münster, Münster, Deutschland; 5 ias-Gruppe, Leipzig, Deutschland; 6 CSL Seqirus GmbH, München, Deutschland; 7 Xcenda GmbH, Hannover, Deutschland; 8 Institut für Labormedizin und Impfzentrum, Klinikum Würzburg Mitte, Standort Juliusspital, Salvatorstr. 7, 97074 Würzburg, Deutschland

**Keywords:** Influenza, Eiadaptation, Weiterentwickelter Influenzaimpfstoff, Real-World-Daten, Impfempfehlung, Influenza, Egg adaptation, Enhanced influenza vaccine, Real-world data, Vaccination recommendation

## Abstract

Auch die Bevölkerung < 60 Jahre ist von einer signifikanten Krankheitslast durch die saisonale Influenza betroffen, einhergehend mit einer hohen wirtschaftlichen Belastung, insbesondere bedingt durch Influenza-assoziierte Produktivitätsverluste der arbeitenden Gesellschaft. Konventionelle eibasierte Influenzaimpfstoffe können aufgrund von Eiadaptation eine beeinträchtigte Impfeffektivität aufweisen. Zellkulturbasierte Influenzaimpfstoffe weisen seltener Antigenanpassungen an das Wirtssystem auf und zeigten über mehrere Saisons eine bessere Effektivität bei 4‑ bis 64-Jährigen gegenüber konventionellen eibasierten Influenzaimpfstoffen unter realen Bedingungen. Der präferenzielle Einsatz von zellkulturbasierten vs. konventionelle Influenzaimpfstoffe könnte zu einer Verringerung der Influenza-bedingten Krankheitslast und wirtschaftlichen Belastung in der deutschen Bevölkerung < 60 Jahre führen.

## Abstract (en)

The population < 60 years of age is also affected by a significant disease burden from seasonal influenza. It carries a high economic burden, mainly driven by influenza-associated productivity losses in the working population. Conventional egg-based influenza vaccines may experience reduced effectiveness due to antigen adaptation in eggs. In contrast, cell-based influenza vaccines are less likely to be affected by antigenic adaptations to the host system and showed better effectiveness in individuals 4–64 years old over several seasons compared to conventional egg-based influenza vaccines under real-world conditions. Preferential use of cell-based influenza vaccines, as opposed to conventional egg-based vaccines, could reduce the burden of influenza-related symptoms on individuals and alleviate the economic impact on the German population < 60 years of age.

In der Saison 2022/23 war Influenza eine der häufigsten laborbestätigten Atemwegserkrankungen in Deutschland, nachdem insbesondere in der Saison 2020/21 aufgrund der Maßnahmen zur Eindämmung der COVID-19-Pandemie relativ wenige laborbestätigte Influenzafälle erfasst wurden [1, 2]. Die Saison 2022/23 war durch einen frühen Beginn und frühen Höhepunkt in Kalenderwoche (KW) 50/2022 gekennzeichnet [1]. Das Ausmaß der Influenzawelle war mit 292.625 laborbestätigten Influenzafällen, 42.562 Hospitalisierungen und 1028 Todesfällen mit Influenzainfektion, die KW 40/2022 bis 21/2023 an das Robert Koch-Institut (RKI) gemeldet wurden, vergleichbar mit schweren Saisons vor der COVID-19-Pandemie.

## Info

Die vergleichsweise wenigen Todesfälle nach laborbestätigter Influenza im epidemiologischen Zusammenhang mit der COVID-19-Pandemie spiegeln nicht die tatsächliche Bedeutung von Morbidität und Mortalität der Influenza wider. Eine bessere Beschreibung der Mortalität bietet die vom RKI ermittelte Exzessmortalität, wobei die höchsten Schätzwerte der letzten 30 Jahre bei 25.100 Todesfällen in der schweren Saison 2017/18 lagen (konservative Schätzung) [3].

Jährlich infizieren sich ca. 5–20 % der Bevölkerung während der saisonalen Influenzawelle [4]. Personen ≥ 60 Jahre sind aufgrund von chronischen Erkrankungen und Immunseneszenz besonders stark von Influenza-bedingten Hospitalisierungen und Todesfällen betroffen [2]. Ein signifikanter Anteil an Infektionen und assoziierten Konsultationen wird jedoch auch bei Personen < 60 Jahre verzeichnet [2, 4]. Vor allem Kinder weisen eine hohe Morbidität und relevante Mortalität auf [5].

## Krankheitslast bei Erwachsenen < 60 Jahre

Die 35- bis 59-Jährigen machten in den Saisons 2016/17 bis 2018/19 einen Großteil (30–38 %) der ans RKI gemeldeten laborbestätigten Influenzafälle aus [2]. Erwachsene < 60 Jahre tragen insbesondere durch Influenza-bedingte Arbeitsunfähigkeit zur wirtschaftlichen Belastung bei [6]: Arbeitsausfälle erwachsener Patienten mit klinisch diagnostizierter Influenza bilden den Großteil der gesellschaftlichen Gesamtkosten in Deutschland [7]. Zudem kann die hohe Krankheitslast bei Kindern zu Arbeitsausfällen bei pflegenden Eltern führen [5] und den Absentismus und damit verbundene Kosten durch Produktivitätsverluste signifikant verstärken.

## Krankheitslast bei Kindern und Jugendlichen

Kinder und Jugendliche weisen eine hohe Infektionsrate auf und sind, anders als bei COVID-19, wesentliche Überträger der Influenza auf Erwachsene und Senioren [4, 8]. Insbesondere Kleinkinder haben ein erhöhtes Risiko für schwere Verläufe. Sie sind, neben der älteren Bevölkerung, am häufigsten von Influenza-bedingten Hospitalisierungen betroffen. Diese werden größtenteils durch Folgekomplikationen (z. B. sekundäre bakterielle Lungenentzündung, akute Mittelohrentzündung und Sinusitis) verursacht [4, 8], die mit tödlichen Krankheitsverläufen einhergehen können. Daten zur Mortalität durch Influenza bei Kindern in Deutschland sind dennoch begrenzt. Im Gegensatz zu Erwachsenen < 60 Jahre ist bei Kindern und Jugendlichen der Großteil der gesellschaftlichen Influenza-assoziierten Gesamtkosten auf direkte Kosten für Arztbesuche und Hospitalisierungen zurückzuführen [7]. Kosten durch Absentismus pflegender Eltern werden dabei nicht berücksichtigt.

## Influenzaprävention in der Bevölkerung < 60 Jahre

Die Ständige Impfkommission (STIKO) empfiehlt eine jährliche Standardimpfung gegen Influenza für alle Personen ≥ 60 Jahre in Deutschland [9]. Für Personen < 60 Jahre wird eine Impfung für Schwangere, Personen ≥ 6 Monate mit erhöhter gesundheitlicher Gefährdung infolge einer Grunderkrankung, Bewohner von Alters- und Pflegeheimen, Betreuende von Risikopersonen sowie Personen mit beruflichem Risiko (z. B. medizinisches Personal, Einrichtungen mit umfangreichem Publikumsverkehr) empfohlen.

Im Gegensatz zur STIKO empfiehlt die Sächsische Impfkommission (SIKO) bereits seit 2010 die jährliche Standardimpfung gegen Influenza für Kinder ab vollendetem 6. Lebensmonat, Jugendliche und Erwachsene [10]. Die SIKO-Empfehlung ist öffentliche Impfempfehlung im Bundesland Sachsen. Die obersten Gesundheitsbehörden aller Länder empfehlen Influenzaimpfungen altersunabhängig über die STIKO-Empfehlung hinaus [11].

### Impfquoten bei Personen < 60 Jahre mit Impfempfehlung

Abrechnungsdaten der Kassenärztlichen Vereinigungen, die 85 % der Bevölkerung erfassen, sowie Daten aus Befragungen von Klinikpersonal aus der Saison 2020/21 ergaben, dass bundesweit nur 39 % der Erwachsenen mit impfrelevanten Grunderkrankungen, 23 % der Schwangeren und etwa 58 % des medizinischen Personals geimpft wurden [12, 13].

## Impfoptionen für Personen < 60 Jahre

Bei den konventionellen, in Deutschland verfügbaren Influenzaimpfstoffen handelt es sich um quadrivalente inaktivierte, nicht adjuvantierte Impfstoffe auf Eibasis in Standarddosierung (QIVe), die für Personen ≥ 6 Monate zugelassen sind [14]. Speziell für Kinder und Jugendliche (2 bis 17 Jahre) ist zudem ein nasaler, eibasierter lebend-attenuierter Impfstoff (LAIV) verfügbar. LAIV wird durch die STIKO in der genannten Altersgruppe gleichwertig zu den konventionellen Impfstoffen empfohlen [9]. Ausnahme sind Situationen, in denen eine Injektion konventioneller Impfstoffe zu Komplikationen führen könnte (z. B. Gerinnungsstörungen oder Spritzenphobie); hier sollte LAIV präferenziell eingesetzt werden. Seit November 2017 empfiehlt die STIKO ausschließlich quadrivalente Impfstoffe, die Antigene jeweils für Influenza A(H1N1), A(H3N2), B(Victoria) und B(Yamagata) enthalten [15].

In Deutschland ist für Personen ≥ 2 Jahre auch ein quadrivalenter zellkulturbasierter Impfstoff (QIVc) zugelassen [14]. Influenzaimpfstoffe wurden weiterentwickelt, um die mitunter geringe Effektivität der konventionellen Impfstoffe zu erhöhen [15]. Dies erfolgt unter anderem entweder über eine „stärkere Aktivierung von humoraler und zellulärer Immunität“ und/oder über eine „konstantere Antigenität“.

## Impfeffektivität (VE) saisonaler Influenzaimpfstoffe

Schätzungen des RKI zur VE gegen laborbestätigte Influenza in Deutschland in den Saisons 2012/13 bis 2019/20 zeigten erhebliche Schwankungen zwischen den Saisons, insbesondere bei dem A(H3N2)-Stamm [16]. Die Ergebnisse einer globalen Metaanalyse (Saisons 2004 bis 2015) von 56 Studien zeigten, dass die VE gegen A(H3N2) sowohl in der pädiatrischen (43 %) als auch in der erwachsenen Kohorte (≤ 60 und > 60 Jahre: 35 % bzw. 24 %) gegenüber der VE gegen A(H1N1) und den B‑Linien deutlich unterlegen war (62–73 % bzw. 54–63 %) [17].

Interimsdaten aus 6 europäischen Studien in der Saison 2022/23 ergaben eine VE jeweils verfügbarer Influenzaimpfstoffe von 2–44 % gegen A(H3N2), 28–46 % gegen A(H1N1) und 64–85 % gegen Influenza B [18].

### Info

Bei der VE saisonaler Influenzaimpfstoffe ist zu beachten, dass diese nicht nur von der Passgenauigkeit zwischen Impfstoff- und zirkulierenden Viren abhängig ist, sondern auch vom Alter des Impflings, dessen Immunkompetenz, früheren Infektionen bzw. Impfungen, der Art des verwendeten Impfstoffs als auch der Pathogenität des (dominant) zirkulierenden Virus [15].

## Nachteile eibasierter Influenzaimpfstoffe

Die Impfstoffherstellung auf Eibasis hat Nachteile, wie z. B. die begrenzte Produktionskapazität, verlängerte Produktionszeit und selten auch allergische Reaktionen auf aus Eiern gewonnenen Proteinen [19]. Des Weiteren können die Isolierung und Vermehrung von humanen Influenzaviren in Eiern zu einer „Eiadaptation“ führen, welche die Effektivität von eibasierten Influenzaimpfstoffen potenziell beeinträchtigt [19, 20]. Eiadaptation kann sowohl bei Influenza-A- als auch -B-Viren auftreten, jedoch ist sie bei A(H3N2)-Viren am häufigsten [21]. Insbesondere Eiadaptation bei Impfviren des Stamms A(H3N2) zeigen negative Auswirkungen auf die VE [20].

### Info

Influenzaviren besitzen eine hohe Mutationsrate, weshalb bei Vermehrung in einem aviären Wirt (Hühnerei) die am besten an das Wirtssystem angepassten Virusvarianten selektiert werden (*Eiadaptation*). Eiadaptation führt zu genetischen Unterschieden zwischen eingesetzten Saatviren und in Eiern produzierten Impfviren durch Mutationen an der Rezeptorbindestelle des Hämagglutinin (HA) und ist wiederum eine wichtige Ursache für antigenes Mismatch von Impfviren und zirkulierenden Viren [19, 20].

## Zellkulturbasierte Influenzaimpfstoffe

Um eine Eiadaptation der Viren zu vermeiden, werden zellkulturbasierte Impfstoffe in Säugetierzellkulturen (Madin Darby Canine Kidney[MDCK]-Zellen) produziert [15, 21]. Auch die zellkulturbasierte Herstellung kann zu Antigenanpassungen an das Wirtssystem führen, Mutationen im HA-Antigen werden dabei aber seltener beobachtet als bei der Vermehrung in Hühnereiern [21]. Dies kann zu einer besseren VE vs. eibasierte Impfstoffe führen.

In Deutschland wurden trivalente zellkulturbasierte Impfstoffe seit der Zulassung vor mehr als 10 Jahren verwendet. Die quadrivalente Formulierung wurde im Dezember 2018 zugelassen [14] und wird seit der Saison 2019/20 in Deutschland verimpft. In Europa ist QIVc ab 2 Jahren zugelassen (Tab. 1). Eine multizentrische, randomisiert kontrollierte Phase-3-Studie (RCT) zur Wirksamkeit von QIVc vs. Nicht-Influenzaimpfstoff bei Kleinkindern (6 bis 47 Monate) über mehrere Saisons läuft derzeit (NCT03932682). Immunogenitäts- und Sicherheitsdaten zu QIVc vs. QIVe der Saison 2019/20 zeigten, dass QIVc bei Kleinkindern gut verträglich und QIVe nicht unterlegen war [22]. Die Sicherheit von QIVc wurde auch speziell bei Schwangeren (inklusive 1. Trimester, *n* = 665) im Alter von 18 bis 46 Jahren in einem über 3 Saisons (2017 bis 2020) laufenden US-Expositionsregister untersucht, in dem keine Sicherheitsbedenken festgestellt wurden [23].

**Table 1:** 

Land	Influenzaimpfstoffe				Quelle
	QIVe	LAIV	QIVc	QIVr^a^	
*Deutschland*	Empfohlen und verfügbar für Pers. **≥** **6** **M.** Erstattet für RGr ≥ 6 M. oder ggf. als Satzungsleistung	Verfügbar für Pers. **2 bis 17** **J.** Erstattet für RGr 2 bis 17 J.	Empfohlen und verfügbar für Pers. **≥** **2** **J.** Erstattet für RGr ≥ 2 J. oder ggf. als Satzungsleistung	NA	[9, 37]
*Italien*	Verfügbar für Pers **≥** **6** **M.** Erstattet für: – Pers. 6 M. bis 6 J. – Pers. ≥ 60 J. – RGr 7 bis 59 J.	Verfügbar für Pers. **2 bis 17** **J.** Erstattet für: – Pers. 2 bis 6 J. – RGr 7 bis 17 J.	Verfügbar für Pers. **≥** **2** **J.** Erstattet für: – Pers. 2 bis 6 J. – Pers. ≥ 60 J. – RGr 7 bis 59 J.	NA	[35]
*Österreich*	Verfügbar für Pers. **≥** **6** **M.** Kostenfrei für: – Pers. 6 M. bis 17 J.	Verfügbar für Pers. **2 bis 17** **J.** (bevorzugte Empfehlung) Kostenfrei für: – Pers. 2 bis 17 J.	Verfügbar für Pers. **≥** **2** **J.**	NA	[33]
*Spanien*	Verfügbar für Pers. **≥** **6** **M.** Erstattet für: – Pers. 6 M. bis 5 J. – Pers. ≥ 60 J. – RGr 6 bis 59 J.	Verfügbar für Pers. **2 bis 17** **J.** Erstattung abhängig von Region	Verfügbar für Pers. **≥** **2** **J.** Erstattet für: – Pers. 2 bis 5 J. – Pers. ≥ 60 J. – RGr 5 bis 59 J.	Begrenzt verfügbar für Pers. **≥** **18** **J.**	[36]
*USA*	Verfügbar für Pers. **≥** **6** **M.**	Verfügbar für Pers. **2 bis 49** **J.**	Verfügbar für Pers. **≥** **6** **M.**	Verfügbar für Pers. **≥** **18** **J.** Erstattet für: – Pers. ≥ 65 J. (bevorzugte Empfehlung mit QIV-HD und aQIV)	[32]
*England*	Verfügbar für Pers. **≥** **6** **M.** Erstattet für: – RGr und berechtigte Jahrgänge 6 M. bis 17 J., wenn QIVc nicht verfügbar ist – RGr 18 bis 64 J., wenn QIVc oder QIVr nicht verfügbar sind	Verfügbar für Pers. **2 bis 17** **J.** Erstattet für: – RGr und berechtigte Jahrgänge 2 bis 17 J.	Verfügbar für Pers. **≥** **6** **M.** Erstattet für: – RGr 6 M. bis 2 J. (Off-Label)^b^ – RGr und berechtigte Jahrgänge 2 bis 17 J., wenn LAIV kontraindiziert oder ungeeignet ist – RGr 18 bis 64 J. (bevorzugte Empfehlung mit QIVr) – Pers. ≥ 65 J., wenn aQIV, QIV-HD oder QIVr nicht verfügbar sind	Verfügbar für Pers. **≥** **18** **J.** Erstattet für: – RGr 18 bis 64 J. (bevorzugte Empfehlung mit QIVc) – Pers. ≥ 65 J. (bevorzugte Empfehlung mit QIV-HD und aQIV)	[38]

Speziell für ältere Erwachsene ≥ 60 bzw. ≥ 65 J. stehen in den meisten Ländern noch QIV-HD bzw. aQIV zur Verfügung [2]

*aQIV* adjuvantierter quadrivalenter Influenzaimpfstoff, J. Jahre, *LAIV* lebend-attenuierter Influenzaimpfstoff, *M.* Monate, *Pers.* Personen, *QIVc* zellkulturbasierter QIV, *QIVe* eibasierter QIV, *QIV-HD* hoch dosierter QIV, *QIVr* rekombinanter QIV, *RGr* Risikogruppen, *USA* Vereinigte Staaten von Amerika

^a^QIVr ist seit November 2020 für Personen ≥ 18 J. in der Europäischen Union zugelassen [14], jedoch noch nicht in Deutschland verfügbar. Bei der Herstellung rekombinanter Impfstoffe wird das HA-Antigen rekombinant über einen Baculovirusvektor in Insektenzellkultur produziert [15]

^b^Zulassungserweiterung für Personen ≥ 6 M. wurde im Oktober 2023 erteilt

## Effektivität des saisonalen zellkulturbasierten Impfstoffs vs. konventionelle eibasierte Impfstoffe

In Beobachtungsstudien wird die VE unter realen Bedingungen (Real-World-Daten [RWD]) gemessen. RWD sind insbesondere für Influenzaimpfstoffe relevant, um die schwankende VE über mehrere Saisons und in verschiedenen Subgruppen, bedingt durch Virus- und Wirtsfaktoren (z. B. Antigendrift, Eiadaptation, Alter [Immunseneszenz], Komorbiditäten, Impfstatus), zu messen sowie seltene Nebenwirkungen zu erfassen [2]. RWD sind somit eine wertvolle Ergänzung zu Daten aus zulassungsrelevanten RCTs und geben Aufschluss darüber, ob ein Impfstoff unter realen Bedingungen effektiv ist.

Im Folgenden werden RWD aus retrospektiven Kohorten- und Fall-Kontroll-Studien zur VE von QIVc vs. QIVe in den US-Saisons 2017/18 bis 2019/20 dargestellt. In der Saison 2017/18 war A(H3N2) in den Vereinigten Staaten von Amerika (USA) dominant, während in der Saison 2018/19 A(H3N2) und A(H1N1) und in der Saison 2019/20 A(H1N1) und B(Victoria) kozirkulierten [24].

### Effektivität von QIVc vs. QIVe bei der Prävention Influenza-bedingter Endpunkte bei Personen ≥ 4 Jahre über mehrere konsekutive Saisons

Eine Kohortenstudie in der Saison 2017/18 bei Personen ≥ 4 Jahre (*n* = 1.353.862) ergab insgesamt eine relative Impfeffektivität (rVE) von QIVc vs. QIVe bei der Prävention von Influenza-ähnlicher Erkrankung (ILI) von 36,2 % (95 %-Konfidenzintervall [KI]: 26,1–44,9) [25]. Bei der Prävention ambulanter und stationärer medizinischer Behandlungen lag die rVE von QIVc vs. QIVe in 2 Kohortenstudien der Saisons 2018/19 (*n* = 10.126.333) bzw. 2019/20 (*n* = 5.625.478) bei 7,6 % (95 %-KI: 6,5–8,6) bei ≥ 4-Jährigen bzw. 9,5 % (95 %-KI: 7,9–11,1) bei ≥ 18-Jährigen [26, 27]. Alle Studien zeigten eine statistisch signifikant höhere rVE bei < 65-Jährigen. Die rVE von QIVc vs. QIVe bei der Prävention von Influenza-bedingten Hospitalisierungen/Besuchen der Notaufnahme bei 4‑ bis 64-Jährigen wurde in 3 Kohortenstudien in den Saisons 2017/18 (*n* = 3.080.510), 2018/19 (*n* = 3.727.890) und 2019/20 (*n* = 5.065.326) analysiert [28–30]. Alle 3 Studien ergaben eine statistisch signifikant höhere rVE von QIVc vs. QIVe (2017/18: 14,4 % [95 %-KI: 8,8–19,6], 2018/19: 6,5 % [95 %-KI: 0,1–12,5], 2019/20: 5,3 % [95 %-KI: 0,5–9,9]).

Die rVE von QIVc vs. QIVe bei der Prävention testbestätigter Influenza wurde in einer Fall-Kontroll-Studie über die Saisons 2017/18 bis 2019/20 bei 4‑ bis 64-Jährigen (*n* = 99.610) analysiert [31]. QIVc war über alle 3 Saisons statistisch signifikant effektiver mit rVEs in Höhe von 14,8 % (95 %-KI: 7,0–22,0) (Saison 2017/18), 12,5 % (95 %-KI: 4,7–19,6) (Saison 2018/19) und 10,0 % (95 %-KI: 2,7–16,7) (Saison 2019/20).

## Influenzaimpfempfehlungen und -optionen in ausgewählten Ländern

Manche Länder (z. B. Österreich, USA) empfehlen eine Influenzaimpfung für alle Personen ≥ 6 Monate [32, 33]. In anderen Ländern (z. B. England, Italien, Spanien) wird die Impfung wie in Deutschland nur für bestimmte Personengruppen ≥ 6 Monate empfohlen [34–36]. Die Tab. 1 gibt einen Überblick über die aktuellen Verfügbarkeiten und bevorzugte Empfehlungen von Influenzaimpfstoffen in ausgewählten Ländern.

QIVc ist derzeit in allen analysierten Ländern für Personen ≥ 2 Jahre bzw. ≥ 6 Monate (USA, Vereinigtes Königreich [seit Oktober 2023]) zugelassen und verfügbar und erhielt in England eine bevorzugte Empfehlung für Personen mit definierter Indikation im Alter von 18 bis 64 Jahren. In Deutschland wird QIVc bei Personen mit Indikation für eine Impfung gleichwertig mit den für die entsprechenden Altersgruppen verfügbaren eibasierten Impfstoffen empfohlen.

## Diskussion

Influenza ist eine der häufigsten Atemwegserkrankungen in Deutschland mit einer signifikanten Krankheitslast in der Bevölkerung. Aufgrund der hohen Morbidität und relevanten Mortalität in der Bevölkerung < 60 Jahre und den damit verbundenen direkten und indirekten Kosten wäre eine Ausweitung der Impfempfehlung der STIKO auf gesunde Erwachsene, Kinder und Jugendliche in Deutschland sinnvoll, wie bereits von der SIKO und den obersten Gesundheitsbehörden sowie in anderen Ländern (z. B. Österreich, USA) umgesetzt. Eine Standardimpfung für Kinder und präferenzielle Impfstoffempfehlung für Indikationsgruppen wird derzeit von der STIKO diskutiert [39].

Ein bedeutender Nachteil der eibasierten Herstellung von Influenzaimpfstoffen ist die Eiadaptation, welche zu einer verringerten VE führen kann, insbesondere gegen Influenza A(H3N2). Ergebnissen eines Konsensusberichts zufolge kann Eiadaptation ein Vakzin-Match um 7–21% und die VE um 4–16% reduzieren [20]. Der zellkulturbasierte und der rekombinante Impfstoff sind derzeit die einzigen zugelassenen Influenzaimpfstoffe, die nicht in Hühnereiern produziert werden, allerdings ist der rekombinante Impfstoff in Deutschland nicht verfügbar (Tab. 1).

### Effektivitäts- und gesundheitsökonomische Daten zum saisonalen zellkulturbasierten Impfstoff

Retrospektive Kohortenstudien der US-Saisons 2017/18 bis 2019/20, die rund 29 Mio. Teilnehmer umfassten, zeigten eine höhere VE von QIVc vs. QIVe bei der Prävention Influenza-bedingter Endpunkte bei Personen im Alter von 4 bis 64 Jahren [25–30]. Eine Limitation dieser Kohortenstudien ist, dass die Influenzafälle nicht laborbestätigt waren. Um Rückschlüsse auf die Effektivität gegen laborbestätigte Influenza ziehen zu können, wurden in den Studien Influenza-spezifische ICD-Codes verwendet, welche einen hohen prädiktiven Wert für laborbestätigte Influenza haben, und in einigen Studien Sensitivitätsanalysen durchgeführt, in denen das Beobachtungsfenster auf die Wochen der höchsten Influenzazirkulation mit der höchsten laborbestätigten Influenzaaktivität beschränkt wurde. Des Weiteren sollten gemäß den klinischen Praxisleitlinien der *Infectious Diseases Society of America *alle Patienten im Krankenhaus mit Influenza-ähnlichen Symptomen auf Influenza getestet werden, was für eine hohe Zuverlässigkeit der Diagnosecodes spricht [29, 30]. Eine Fall-Kontroll-Studie mit ca. 100.000 Personen (4 bis 64 Jahre) in denselben US-Saisons zeigte, dass QIVc auch bei der Prävention von testbestätigter Influenza effektiver war als QIVe [31].

Eine gesundheitsökonomische Evaluation ergab zudem, dass der Einsatz von QIVc vs. QIVe in Deutschland zur Vermeidung von Influenzafällen und Komplikationen und gleichzeitigen Kosteneinsparungen aus gesellschaftlicher Sicht führen und somit kosteneffektiv sein könnte [40].

## Fazit

RWD aus den US-Saisons 2017/18 bis 2019/20 zeigten, dass QIVc bei Personen < 65 Jahre effektiver war als QIVe. Der präferenzielle Einsatz von QIVc könnte auch in Deutschland zur Verringerung der Influenza-bedingten Krankheitslast und zusätzlich der damit assoziierten direkten und indirekten Kosten in der Bevölkerung < 60 Jahre führen.

## Kernaussagen

- Die Bevölkerung < 60 Jahre trägt signifikant zur Krankheitslast und ökonomischen Belastung durch die saisonale Influenza in Deutschland bei, wobei insbesondere Kinder von schweren Krankheitsverläufen betroffen sind sowie relevante Überträger der Influenza auf Erwachsene und Senioren darstellen. Die arbeitende Bevölkerung verursacht aufgrund von eigenen Erkrankungen oder der Pflege erkrankter Kinder hohe Produktivitätsverluste.
- Niedrige Influenzaimpfquoten in Deutschland und die mitunter erheblich schwankende Impfeffektivität verfügbarer Influenzaimpfstoffe zwischen den Saisons führen zu einem suboptimalen Schutz der Bevölkerung vor Infektionen und schweren Verläufen.
- Eiadaptation konventioneller eibasierter Influenzaimpfstoffe kann ein Mismatch von Impfviren und zirkulierenden Viren verursachen und dadurch die Effektivität dieser Impfstoffe beeinträchtigen.
- Zellkulturbasierte Influenzaimpfstoffe sind seltener von Antigenanpassungen an das Wirtssystem betroffen und zeigten eine signifikant bessere Effektivität als konventionelle eibasierte Influenzaimpfstoffe bei der Prävention von (testbestätigten) Influenzaerkrankungen und Influenza-assoziierten Behandlungen unter realen Bedingungen.
- Eine Ausweitung der Impfempfehlung auf alle Altersgruppen und eine präferenzielle Empfehlung zellkulturbasierter vs. konventioneller eibasierter Influenzaimpfstoffe könnte die Influenza-bedingte Krankheitslast und damit assoziierten Kosten in der deutschen Bevölkerung < 60 Jahre reduzieren.

## Interessenkonflikt

B.C. Gärtner: Advisory Board und Honorare für Vorträge für CSL Seqirus, Sanofi, GSK, Moderna, Biontech, Pfizer, Viatris. D. Beier: Advisory Board für CSL Seqirus. G. Gosch: Vortragstätigkeiten und Teilnahme an Advisory Boards: AstraZeneca, CSL Seqirus, GSK, MSD, Pfizer, Sanofi-Aventis. K. Wahle: Vortragstätigkeiten und Teilnahme an Advisory Boards im Auftrag bzw. für die Firmen SPMSD, CSL Seqirus, Pfizer, GSK, Biontech. L.-C. Förster ist bei der CSL Seqirus GmbH angestellt. K.J. Schmidt ist bei der Xcenda GmbH angestellt, die für die Erstellung dieses Manuskripts von der CSL Seqirus GmbH finanziert wurde. T.F. Schwarz: Honorare für Beratung und Vorträge: Alexion, AstraZeneca, Bavarian Nordic, Biogen, Biontech, GSK, Janssen-Cilag, Merck-Serono, Moderna, MSD, Novavax, Pfizer, Roche, Sanofi-Aventis, CSL Seqirus, Synlab, Takeda und va-Q-tec. L. Wendt gibt an, dass kein Interessenkonflikt besteht.
